# One Case of Sudden Isolated Adrenocorticotropic Hormone (ACTH) Deficiency Diagnosed Based on Repeated Hypoglycemic Attacks

**DOI:** 10.7759/cureus.86185

**Published:** 2025-06-17

**Authors:** Tomohide Sato

**Affiliations:** 1 Division of Cardiology, Saiseikai Kumamoto Hospital Cardiovascular Center, Kumamoto, JPN

**Keywords:** cortisol, hashimoto's thyroiditis, hypoglycemic, secondary adrenopause, sudden isolated adrenocorticotropic hormone (acth) deficiency

## Abstract

Our patient is a 28-year-old male who was being treated by a local doctor for Hashimoto's thyroiditis. Four days prior to admission, nausea and diarrhea appeared, and it gradually became difficult for him to eat. The night before admission, his level of consciousness decreased (Japan Coma Scale (JCS) II-20), and he was rushed to the hospital. His other vital signs were stable. After completing a detailed examination, the cause was diagnosed as hypoglycemia with a blood glucose level of 21 mg/dl. After the administration of glucose, he regained consciousness and became alert, allowing him to return home, with the expectation that he would return to the hospital for a follow-up visit at a later date. However, an altered consciousness (JCS I-3) appeared again the next morning. Similar to the previous day, the cause of the altered consciousness was determined to be hypoglycemia. After making a detailed inpatient examination, his early morning fasting serum cortisol level was found to be less than 0.1 μg/dL, and his blood adrenocorticotropic hormone (ACTH) was 3.1 pg/mL, thus indicating decreases in both. After performing rapid ACTH testing, almost no increase in the serum cortisol levels was observed after 30 minutes and 60 minutes following ACTH administration, thus suggesting the presence of adrenal insufficiency. According to a contrast-enhanced CT of the abdomen, atrophy of both adrenal glands was observed; however, there was no macroadenoma or the like according to the contrast-enhanced MRI of the brain. Based on the results of the ACTH continuous load test, triple anterior pituitary stimulation test with corticotropin-releasing hormone (CRH)*, *thyrotropin-releasing hormone (TRH), and gonadotropin-releasing hormone (GnRH),* *growth hormone-releasing peptide-2 (GHRP-2) load test, and insulin hypoglycemic load test, no abnormalities were found in his pituitary functions other than ACTH-cortisol, and no history of trauma or adenoma lesions, leading to a diagnosis of a sudden isolated ACTH deficiency. The patient has remained stable with no hypoglycemic episodes since treatment with hydrocortisone was initiated. Based on the fact that isolated ACTH deficiency is often associated with a complication of autoimmune endocrine disease, an autoimmune mechanism has been speculated. Although the disease is rare, it is an important disease that affects the quality of life (QOL) of patients, and it should therefore be considered when making a differential diagnosis.

## Introduction

Isolated adrenocorticotropic hormone (ACTH) deficiency is a rare endocrine disorder characterized by secondary adrenal insufficiency due to a selective loss of ACTH secretion, with preservation of other pituitary hormones [1]. It can present with non-specific symptoms such as fatigue, nausea, hypotension, and, in severe cases, recurrent fasting hypoglycemia and altered consciousness [2]. The reported prevalence of isolated idiopathic ACTH deficiency is approximately 19.1 per million people, making it an uncommon but clinically significant condition [3]. Although its pathogenesis remains unclear, autoimmune mechanisms have been proposed as a potential etiology, particularly when associated with other autoimmune endocrine diseases such as Hashimoto's thyroiditis or type 1 diabetes mellitus [4]. Due to its nonspecific presentation and potential for delayed diagnosis, clinicians should maintain a high index of suspicion, especially in patients with unexplained hypoglycemia. Herein, we report a case of isolated idiopathic ACTH deficiency diagnosed following repeated hypoglycemic episodes.

## Case presentation

A 28-year-old man presented to our emergency department with impaired consciousness. He had a known history of Hashimoto’s thyroiditis diagnosed at the age of 27 and had been taking levothyroxine sodium hydrate 75 μg daily. His family history was notable for type 2 diabetes in his grandmother. He was a non-smoker and consumed alcohol occasionally. After completing graduate school, he had been employed but later resigned due to severe fatigue and had been unemployed and living at home since then.

Four days before admission, he developed nausea and diarrhea. He was seen by a local physician and was diagnosed with acute gastroenteritis. However, due to worsening appetite, he was unable to maintain adequate oral intake, and by the day before admission, he was nearly fasting. That night, he was found in a drowsy state and transported to the emergency department. His level of consciousness was assessed as Japan Coma Scale (JCS) II-20. His blood glucose level was 21 mg/dL, indicating significant hypoglycemia. After intravenous administration of glucose, his consciousness fully recovered, and he returned home with instructions to follow up the next day.

However, on the following morning, he again exhibited decreased consciousness (JCS I-3) and returned to the hospital. His blood glucose level was 39 mg/dL, again consistent with hypoglycemia. Given the recurrence of hypoglycemia in a young patient without a history of diabetes, he was admitted for further evaluation.

On admission, the patient was alert but appeared slightly drowsy. His vital signs were as follows: body temperature 36.9°C, respiratory rate 12 breaths per minute, blood pressure 127/98 mmHg, pulse rate 94 beats per minute, and oxygen saturation 100% on room air. He was 159 cm tall and weighed 47.0 kg, with a BMI of 18.6. Physical examination revealed no pallor of the palpebral conjunctiva and no pigmentation of the oral mucosa or skin. Superficial lymph nodes were not palpable. The thyroid gland was elastic and firm on palpation but not enlarged. Chest and abdominal examinations were unremarkable. His skin was dry, and both truncal and limb body hair appeared slightly reduced, including pubic hair thinning. No signs of hypogonadism or other endocrinopathies were noted on physical examination.

Laboratory findings are shown in Table 1.

**Table 1:** Blood tests MCV: mean corpuscular volume; MCH: mean corpuscular hemoglobin; MCHC: mean corpuscular hemoglobin concentration; BUN: blood urea nitrogen; AST: aspartate aminotransferase; GOT: glutamic oxaloacetic transaminase; ALT: alanine aminotransferase; GPT: glutamic pyruvate transaminase; LDH: lactate dehydrogenase; ALP: alkaline phosphatase; γ-GTP: gamma-glutamyl transferase; HDL-C: high density lipoprotein cholesterol; LDL-C: low density lipoprotein cholesterol; CRP: c-reactive protein test; HbA1c: glycated hemoglobin; TSH: thyroid stimulating hormone; FT3: free triiodothyronine blood test; FT4: free thyroxine blood test; UIBC: unsaturated iron binding capacity; TIBC: total iron binding capacity; ANA: antinuclear antibody test; Anti-Tg Ab: anti-thyroglobulin antibody; Anti-TPO Ab: anti-thyroid peroxidase antibody; ACTH: adrenocorticotropic hormone

Test	Result	Reference Range
WBC	5500 /μL	4000–10000 /μL
Neutrophils	37.2%	40–70%
Lymphocytes	42.6%	20–40%
Monocytes	4.9%	2–8%
Eosinophils	14.8%	0–6%
Basophils	0.5%	0–1%
RBC	407×10⁴/μL	400–550×10⁴/μL
Hemoglobin	12.3 g/dL	13.0–17.0 g/dL
Hematocrit	32.5%	38–50%
MCV	79.9 fL	80–100 fL
MCH	30.2 pg	27–33 pg
MCHC	37.8 g/dL	32–36 g/dL
Platelets	20.3×10⁴/μL	13–37×10⁴/μL
Total Protein	5.9 g/dL	6.5–8.0 g/dL
Albumin	3.4 g/dL	4.0–5.0 g/dL
Sodium	135 mEq/L	136–145 mEq/L
Potassium	3.9 mEq/L	3.5–5.0 mEq/L
Chloride	104 mEq/L	98–107 mEq/L
Calcium	8.0 mg/dL	8.5–10.5 mg/dL
BUN	8.6 mg/dL	8–20 mg/dL
Creatinine	0.58 mg/dL	0.6–1.1 mg/dL
Glucose	203 mg/dL	70–110 mg/dL
AST (GOT)	39 IU/L	10–40 IU/L
ALT (GPT)	25 IU/L	5–45 IU/L
LDH	184 IU/L	120–240 IU/L
Total Bilirubin	0.51 mg/dL	0.2–1.2 mg/dL
ALP	204 IU/L	100–340 IU/L
γ-GTP	19 IU/L	10–47 IU/L
Amylase	71 IU/L	37–125 IU/L
Total Cholesterol	147 mg/dL	140–199 mg/dL
HDL-C	21 mg/dL	40–80 mg/dL
LDL-C	107 mg/dL	70–139 mg/dL
Triglycerides	73 mg/dL	50–149 mg/dL
CRP	0.7 mg/dL	<0.3 mg/dL
Ammonia (NH3)	46 μg/dL	30–80 μg/dL
HbA1c	5.1%	4.6–6.2%
TSH	9.6831 μIU/mL	0.5–5.0 μIU/mL
FT3	1.97 pg/mL	2.3–4.0 pg/mL
FT4	0.87 ng/dL	0.9–1.7 ng/dL
Iron	57 μg/dL	70–170 μg/dL
UIBC	127 μg/dL	150–300 μg/dL
TIBC	184 μg/dL	250–370 μg/dL
Ferritin	185.7 ng/mL	30–400 ng/mL
ANA	<1:40	<1:40
IgG4	102 mg/dL	4–134 mg/dL
Anti-Tg Ab	509 IU/mL	<40 IU/mL
Anti-TPO Ab	212 IU/mL	<30 IU/mL
Cortisol (AM)	<0.1 μg/dL	6.0–18.4 μg/dL
ACTH	3.1 pg/mL	7.2–63.3 pg/mL
Glucagon	78 pg/mL	50–150 pg/mL
Adrenaline	8 pg/mL	<100 pg/mL
Noradrenaline	378 pg/mL	100–450 pg/mL

Blood tests revealed a normal white blood cell count with a notable eosinophilia of 14.8%. The observed eosinophilia (14.8%) may reflect cortisol deficiency, as cortisol normally suppresses eosinophil counts; eosinophilia can thus be seen in adrenal insufficiency. Hemoglobin was slightly low at 12.3 g/dL, and serum total protein and albumin were mildly decreased. Electrolytes showed sodium at 135 mEq/L and potassium at 3.9 mEq/L. Notably, fasting glucose on admission was 203 mg/dL, likely reactive after glucose administration. Inflammatory markers were not significantly elevated (C-reactive protein (CRP) 0.7 mg/dL). Thyroid function testing showed elevated thyroid-stimulating hormone (TSH) (9.6831 μIU/mL) and low-normal Free Thyroxine 4 (FT4) (0.87 ng/dL), consistent with suboptimal replacement. Early morning cortisol was <0.1 μg/dL, and ACTH was 3.1 pg/mL, both markedly decreased. Further hormonal values, including glucagon, adrenaline, and noradrenaline, were within or near the normal range. Thyroid antibodies, including anti-thyroglobulin antibody and anti-thyroid peroxidase (anti-TPO) antibody, were elevated.

**Figure 1:**
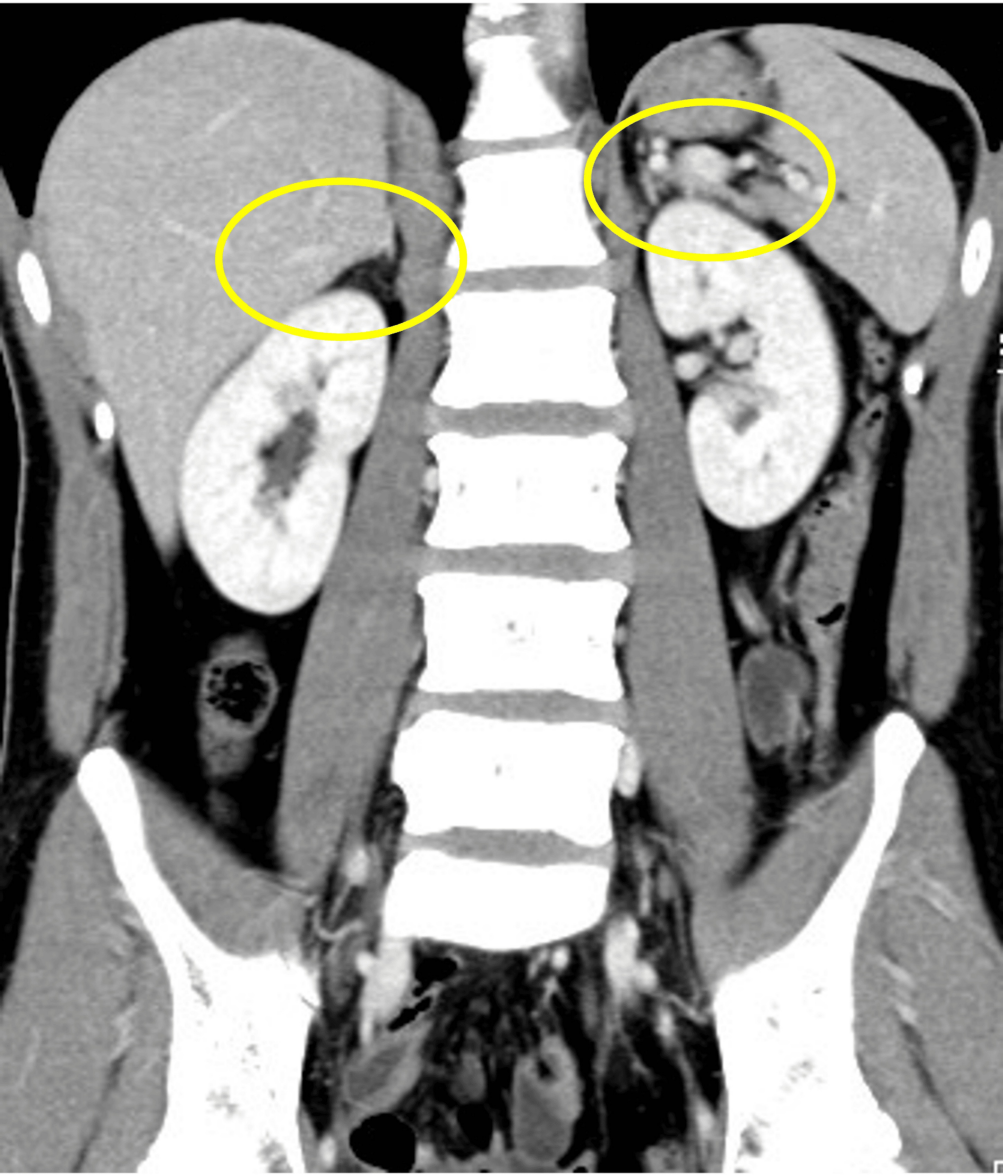
Contrast-enhanced CT scan of the abdomen Coronal view of contrast-enhanced abdominal CT. Both adrenal glands appear atrophic, with no evidence of enlargement or tumor formation.

**Figure 2:**
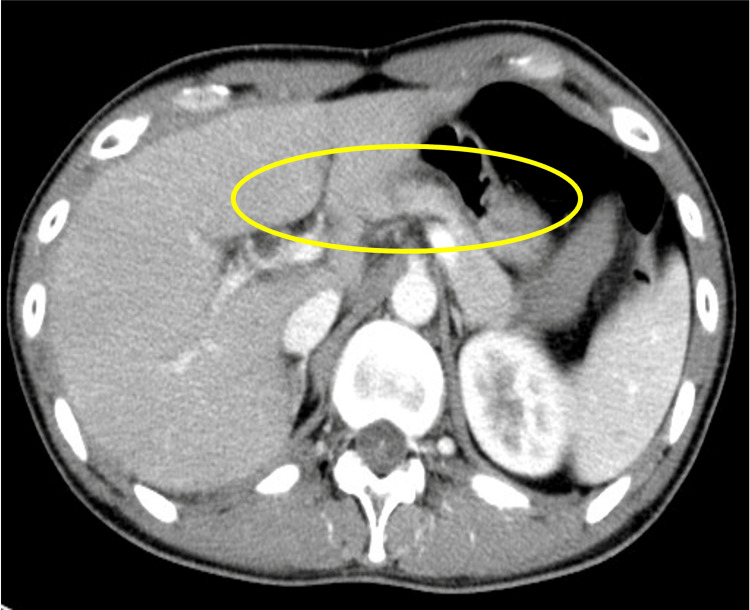
Contrast-enhanced CT scan of the abdomen Axial view of contrast-enhanced abdominal CT. The pancreas is unremarkable, and no abnormal findings are observed in surrounding retroperitoneal structures.

Contrast-enhanced computed tomography (CT) of the abdomen revealed bilateral adrenal gland atrophy without evidence of masses or nodular lesions. In addition, there were no abnormal findings in the pancreas or surrounding retroperitoneal structures (Figures 1, 2). 

**Figure 3:**
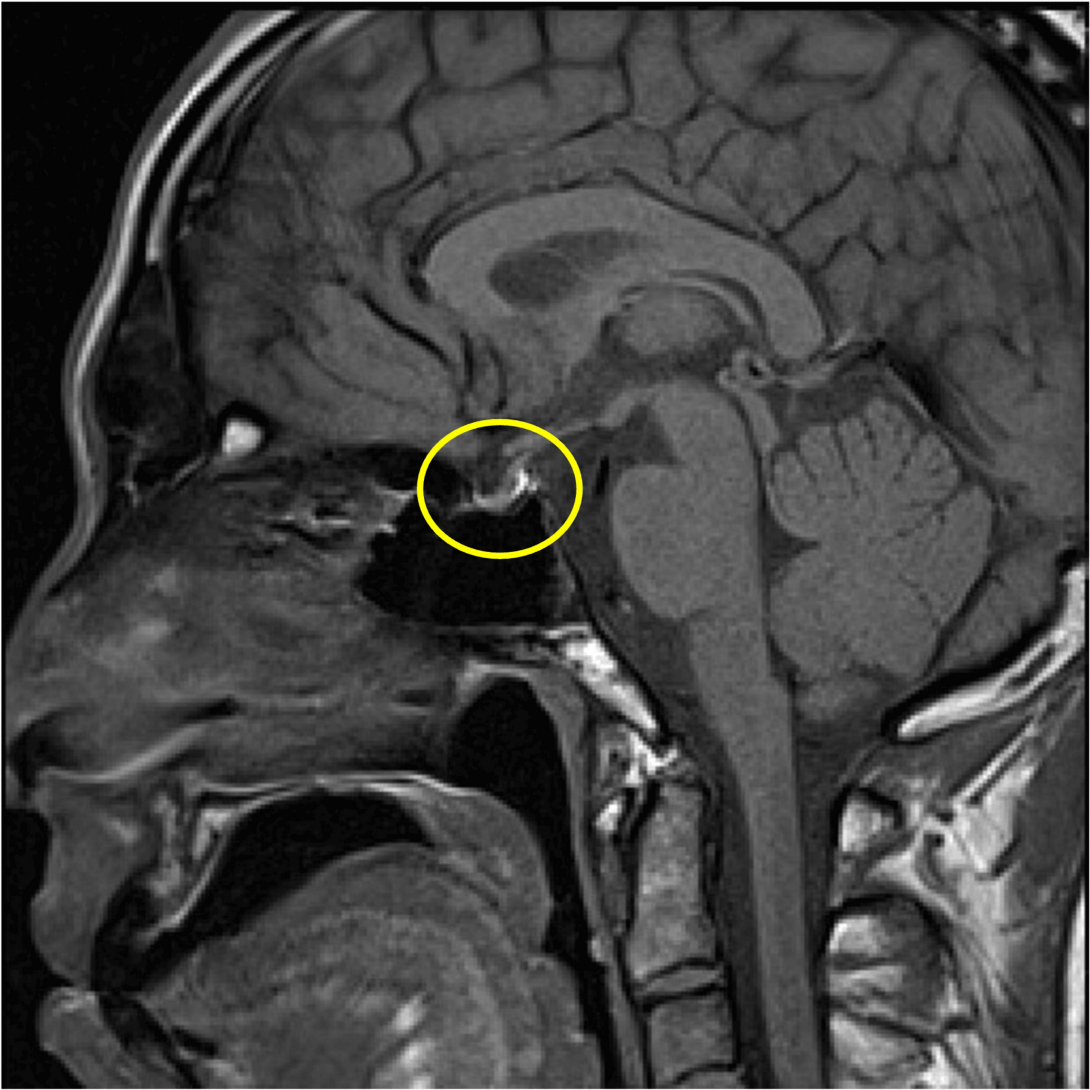
Contrast-enhanced MRI scan of the brain Sagittal T1-weighted MRI of the brain. The pituitary gland appears normal in size and contour. No evidence of swelling or mass lesion is observed.

**Figure 4:**
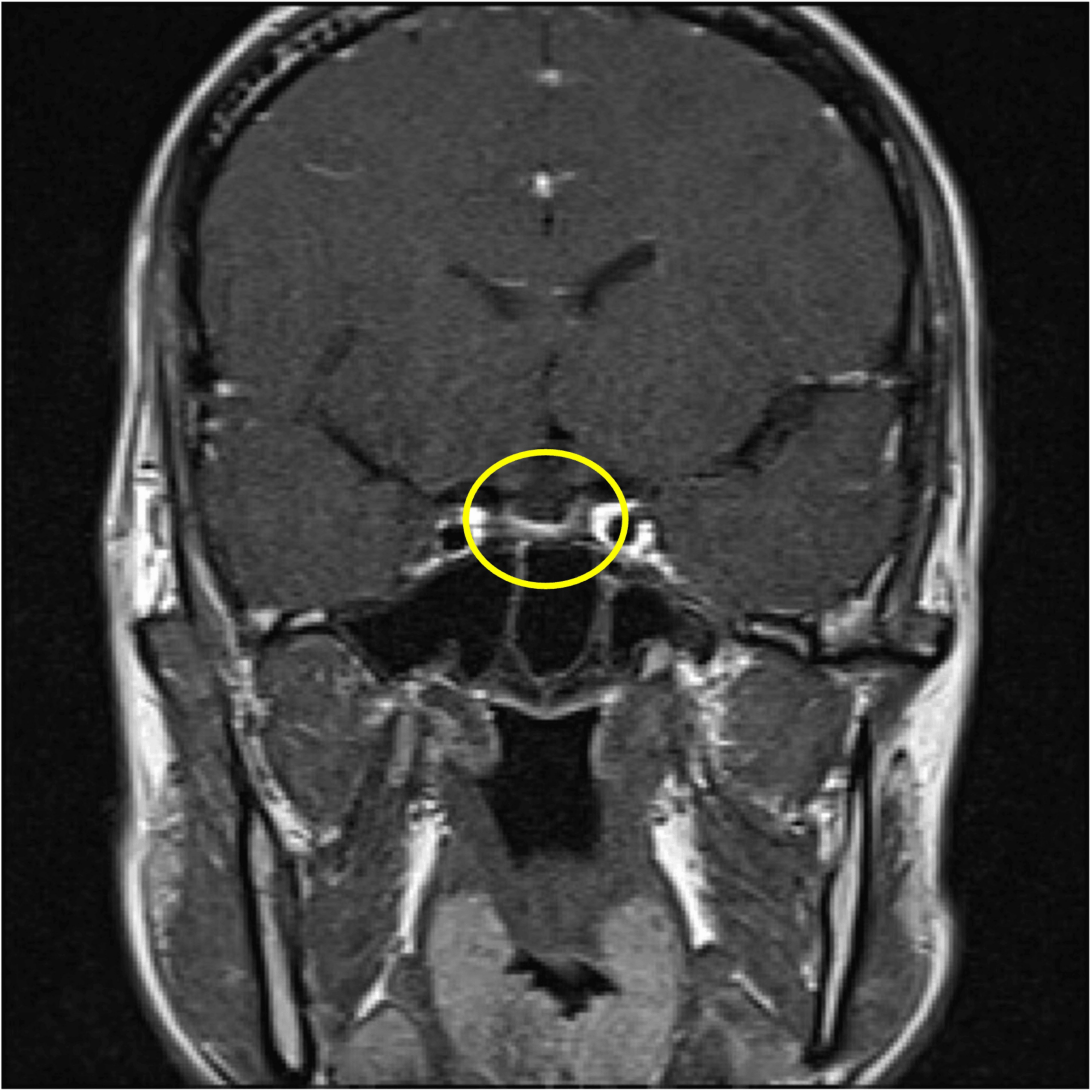
Contrast-enhanced MRI scan of the brain Coronal contrast-enhanced T1-weighted MRI. The pituitary gland shows no signs of enlargement, and no macroadenoma is detected.

**Figure 5:**
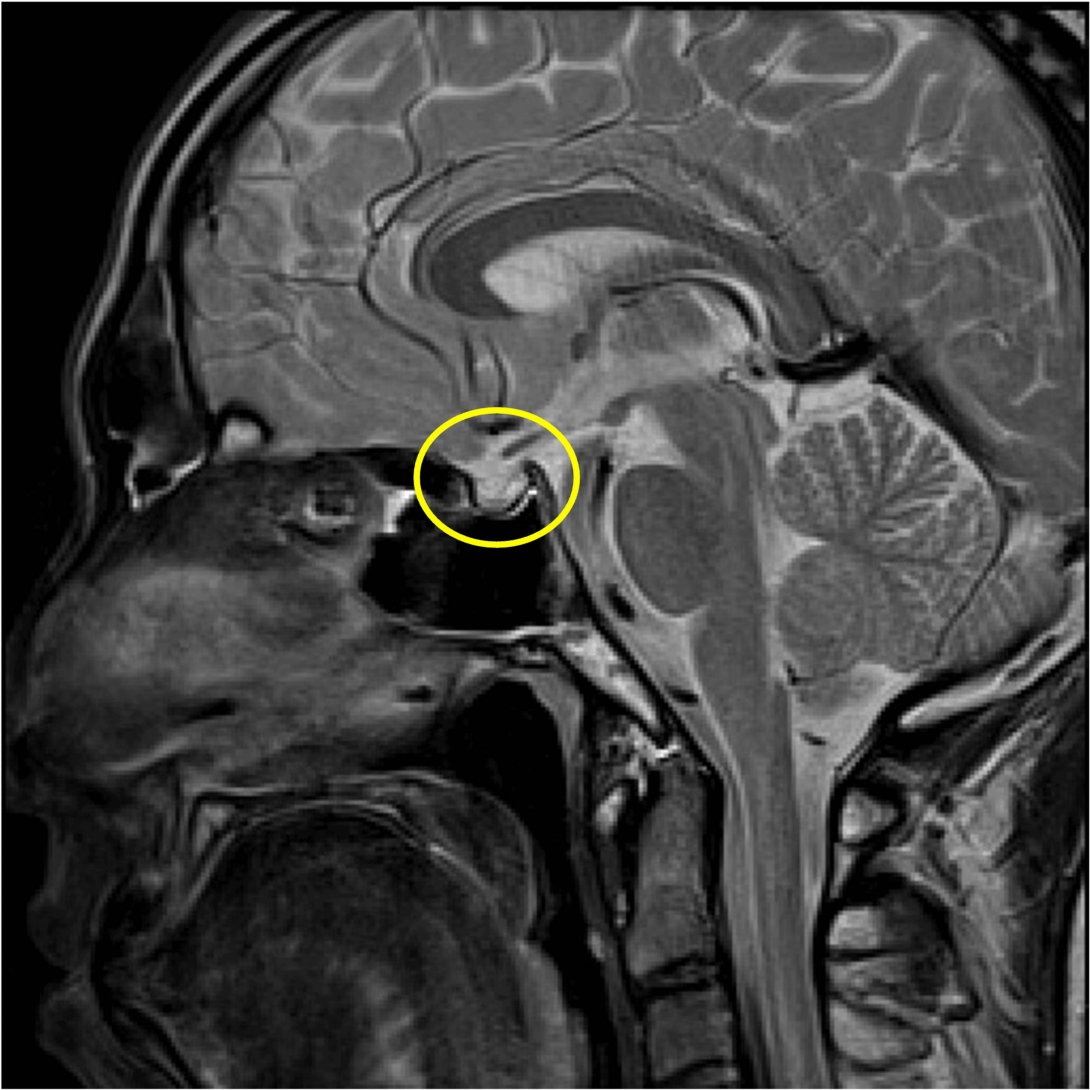
Contrast-enhanced MRI scan of the brain Sagittal T2-weighted MRI. The pituitary gland maintains a normal signal intensity and morphology without mass effect.

**Figure 6:**
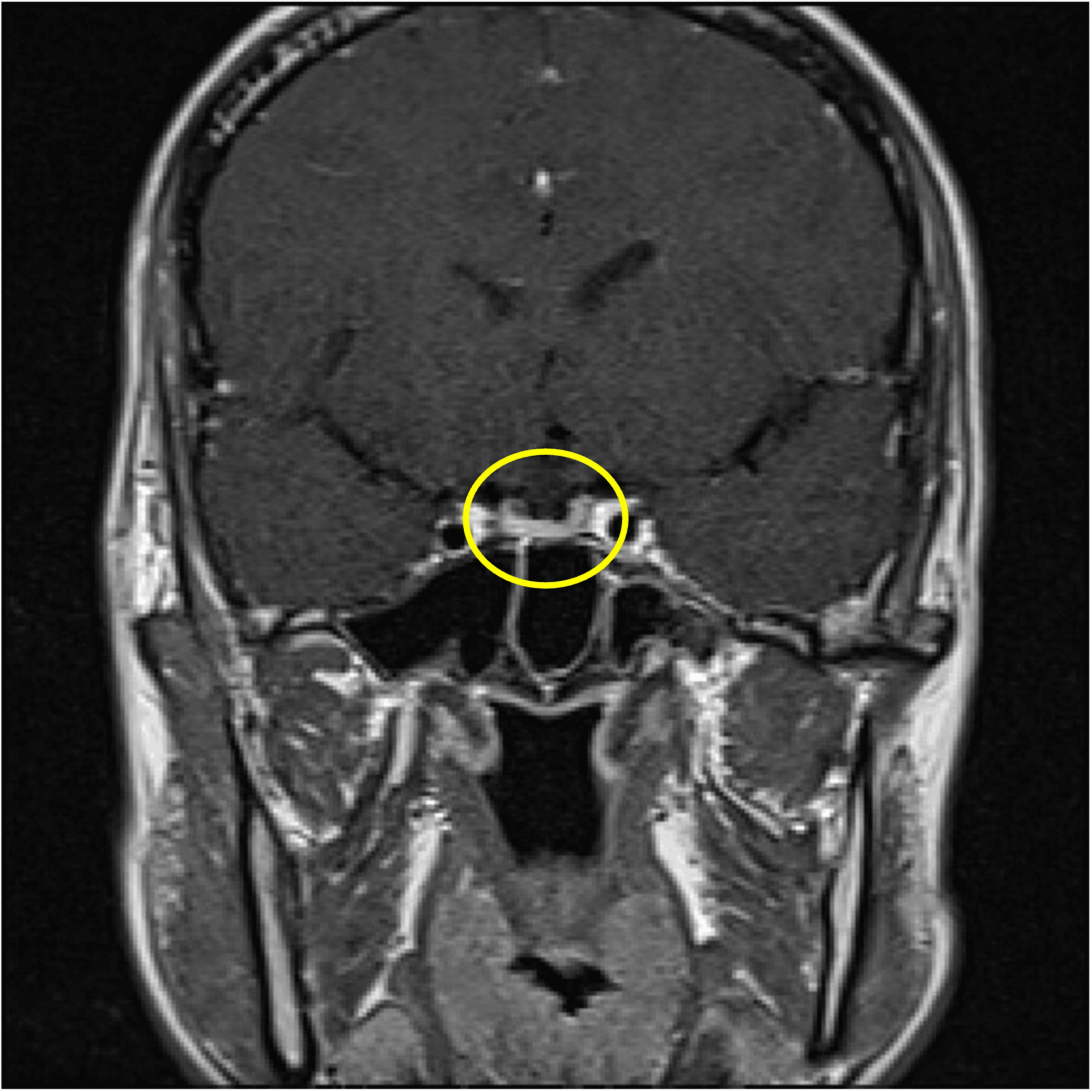
Contrast-enhanced MRI scan of the brain Coronal contrast-enhanced MRI (late phase). The pituitary region reveals no abnormal enhancement or structural distortion, consistent with absence of macroadenoma.

Brain magnetic resonance imaging (MRI), including T1-weighted, T2-weighted, and dynamic sequences, showed no evidence of pituitary macroadenoma or structural abnormalities (Figures 3-6).

Given the very low cortisol and ACTH levels, adrenal insufficiency was suspected. A rapid ACTH stimulation test using 250 μg of tetracosactide acetate was performed (Table 2), revealing almost no increase in cortisol levels at 30 and 60 minutes post-injection.

**Table 2:** Rapid adrenocorticotropic hormone (ACTH) stimulation test results. Rapid ACTH load test: 250 μg of tetracosactide acetate was used. Serum cortisol levels remained below the assay sensitivity threshold (<0.1 μg/dL at baseline, and 0.2 μg/dL at both 30 and 60 minutes post-injection), indicating no adrenal response to exogenous ACTH. This finding supports a diagnosis of secondary adrenal insufficiency.

	Before loading	30 minutes	60 minutes
Cortisol in the blood (μg/dL)	<0.1	0.2	0.2

To rule out insulinoma and other causes of hypoglycemia, a 72-hour fasting test was conducted. Blood samples taken 35 hours after initiation did not show abnormal insulin secretion (Table 3), making insulinoma unlikely.

**Table 3:** Results from the 72-hour fasting test. Results from the 72-hour fasting test: values recorded from blood taken 35 hours after initiation. The study was terminated at 35 hours when the patient's blood glucose dropped to 45 mg/dL. At that time, laboratory evaluation showed that both C-peptide and insulin levels were suppressed (C-peptide: 0.05 ng/mL; insulin: <0.3 μIU/mL), indicating a physiologically appropriate response to hypoglycemia. These findings ruled out endogenous hyperinsulinism, such as insulinoma.

Measurement	Results
C-Peptide	0.05 ng/mL
Insulin	<0.3 μIU/mL

**Figure 7:**
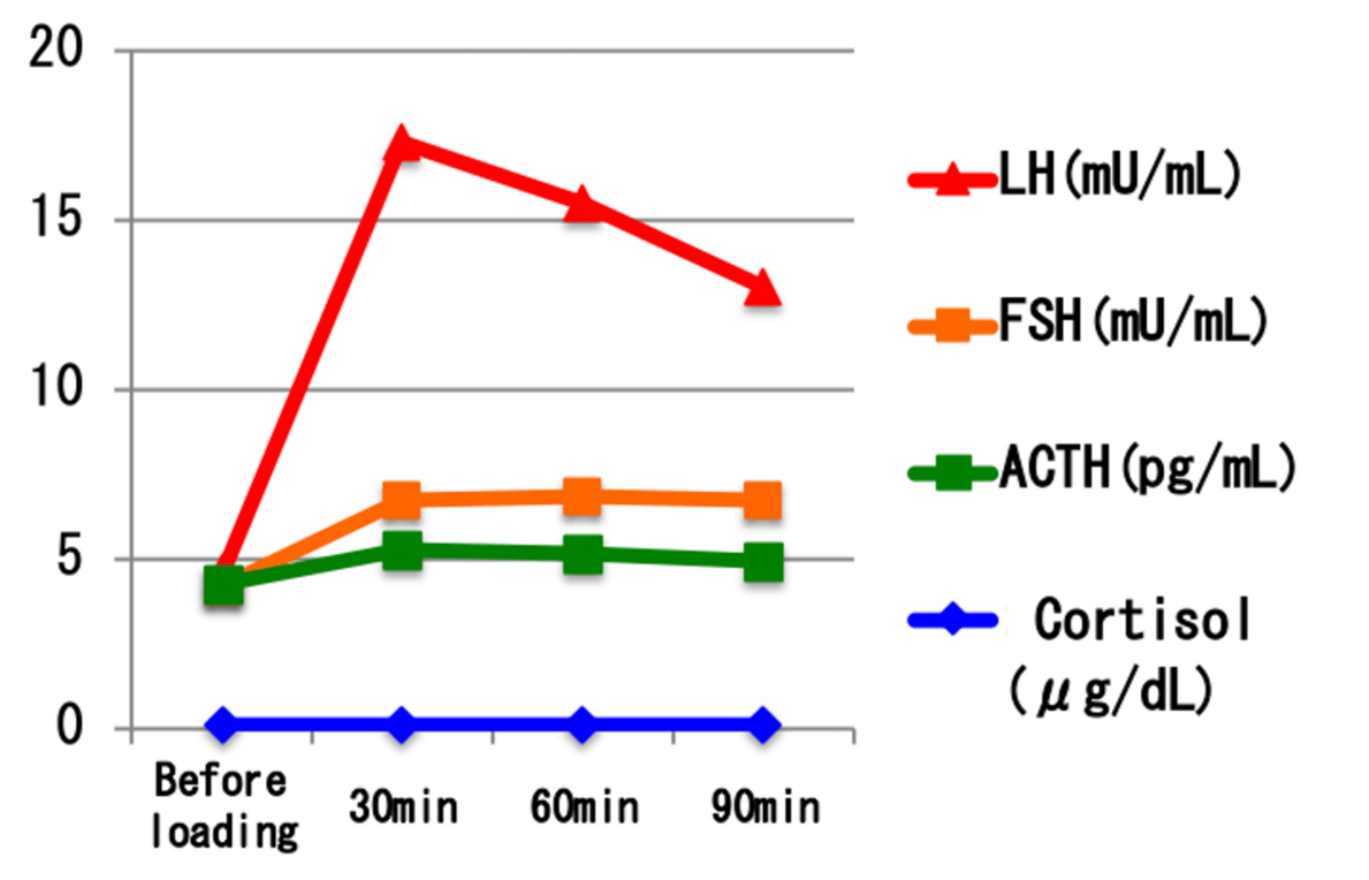
Results of the combined CRH and LH-RH stimulation test Tripartite load test: growth hormone-releasing hormone (GHRH) 100 μg, luteinizing hormone-releasing hormone (LH-RH) 0.1 mg, thyrotropin-releasing hormone (TRH) 0.2 mg were used. Adrenocorticotropic hormone (ACTH; green line) showed minimal elevation after corticotropin-releasing hormone (CRH) administration, and serum cortisol (blue line) remained below the assay’s sensitivity threshold throughout the test, indicating impaired hypothalamic-pituitary-adrenal axis function. Luteinizing hormone (LH; red line) and follicle-stimulating hormone (FSH; orange line) levels increased after LH-RH administration, with LH reaching less than a five-fold rise from baseline, suggesting a suboptimal but present gonadotropin response. Baseline LH and FSH levels were within the normal range.

**Figure 8:**
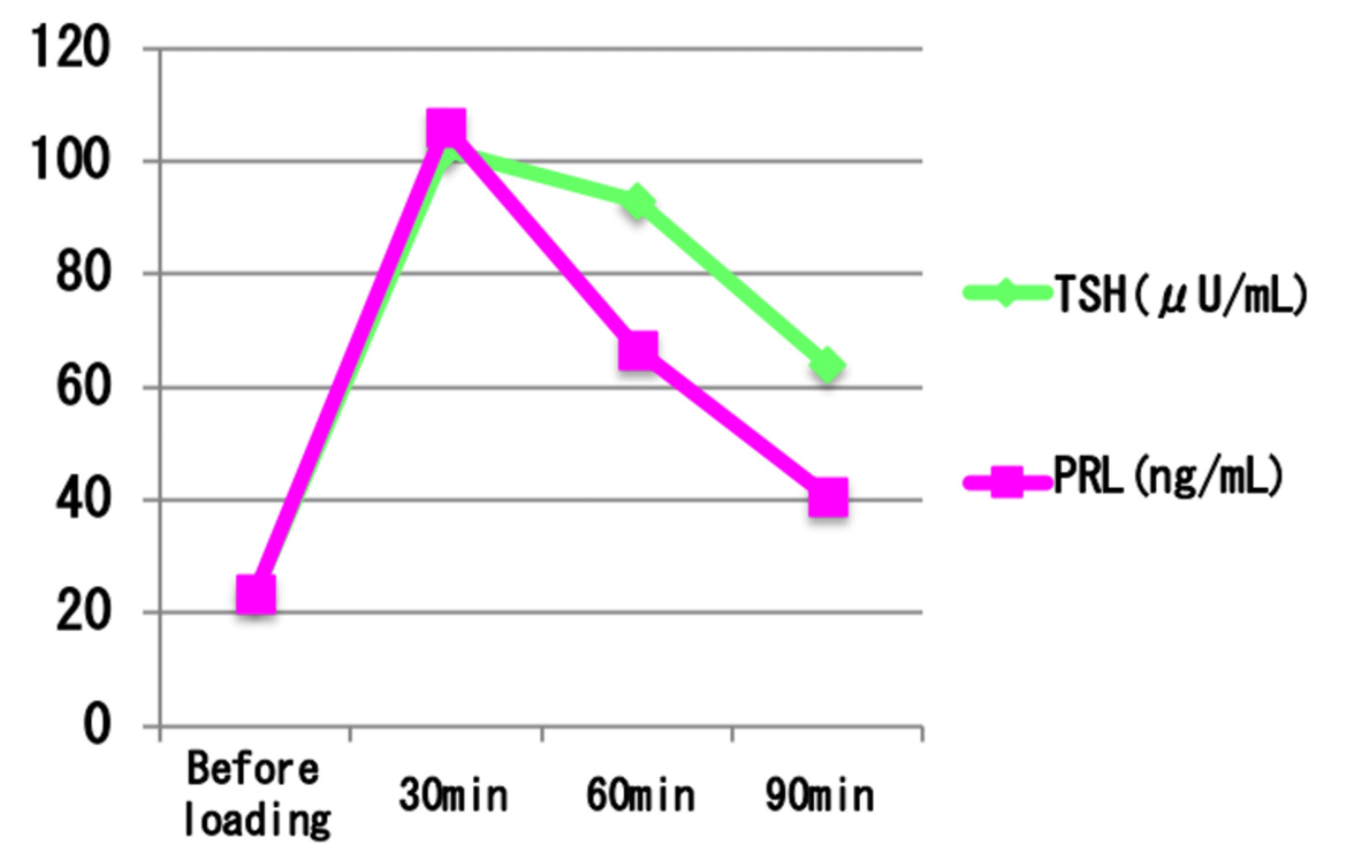
Results of the thyrotropin-releasing hormone (TRH) stimulation test. Tripartite load test: growth hormone-releasing hormone (GHRH) 100 μg, luteinizing hormone-releasing hormone (LH-RH) 0.1 mg, thyrotropin-releasing hormone (TRH) 0.2 mg were used. Thyroid-stimulating hormone (TSH; green line) showed an elevated baseline level and responded further after TRH administration, peaking at 30 minutes. Prolactin (PRL; magenta line) also demonstrated a physiological increase after stimulation. These findings indicate preserved TRH responsiveness in the pituitary-thyroid and pituitary-prolactin axes, although the elevated baseline TSH suggests subclinical or inadequately treated hypothyroidism.

**Figure 9:**
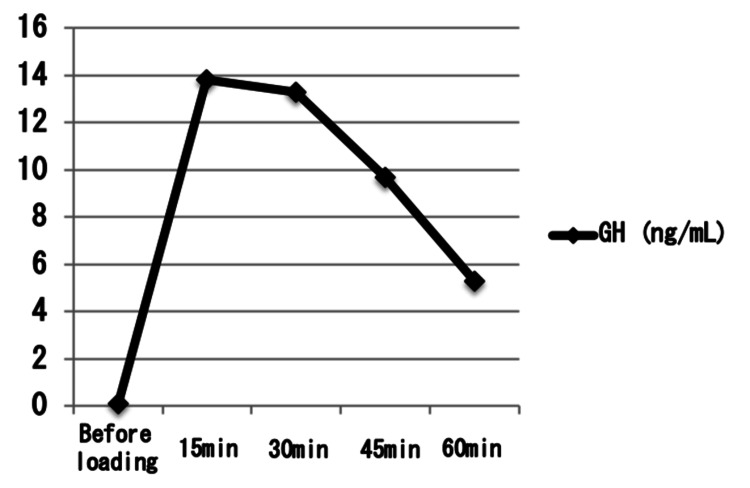
Growth hormone response to the growth hormone-releasing peptide-2 (GHRP-2) stimulation test. GHRP-2 load test: GHRP-2 100 μg was used. Serum growth hormone (GH) levels peaked at 13.8 ng/mL 15 minutes after GHRP-2 administration, exceeding the diagnostic threshold of 9 ng/mL. This response indicates an intact hypothalamic-pituitary-growth hormone axis and is considered a normal physiological reaction.

**Figure 10:**
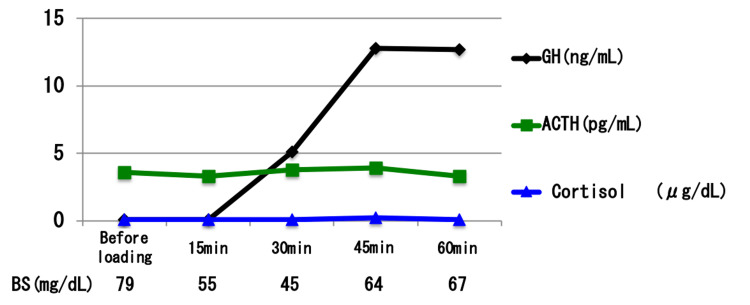
Results of the insulin-induced hypoglycemia test. Insulin hypoglycemic load test: 2.5 units of human insulin were used. In this test, insulin was administered at 0.05 units/kg (lower than the standard 0.1 units/kg) due to suspected adrenal insufficiency. Blood glucose levels decreased to 45 mg/dL at 30 minutes, confirming an effective hypoglycemic stimulus. Although adrenocorticotropic hormone (ACTH; green line) showed a slight increase, the levels remained below the expected physiological response range. Serum cortisol (blue line) remained at or below the assay’s sensitivity limit throughout the test, indicating a blunted adrenal response. These findings are consistent with secondary adrenal insufficiency.

**Figure 11:**
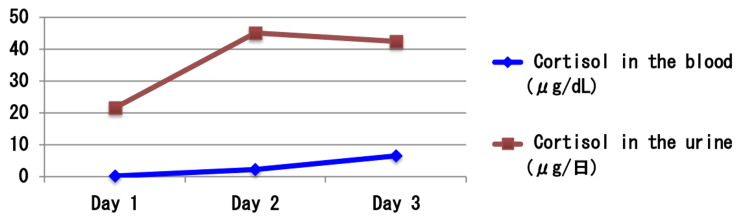
Results of the continuous ACTH stimulation test. Continuous adrenocorticotropic hormone (ACTH) load test: tetracosactide acetate 0.5 mg was used. Cortisol levels in the blood (blue line) and urine (brown line) were monitored over three days during continuous tetracosactide acetate administration. On Day 1, cortisol levels remained low and were difficult to evaluate; however, both serum and urinary cortisol increased slightly over time, indicating a delayed but present adrenal response. These findings suggest residual adrenal responsiveness to ACTH and are consistent with secondary adrenal insufficiency rather than primary adrenal failure.

To further evaluate anterior pituitary function, a series of stimulation tests were performed, including a triple stimulation test (thyrotropin-releasing hormone (TRH) 0.2 mg, luteinizing hormone-releasing hormone (LH-RH)** **0.1 mg, growth hormone-releasing hormone (GHRH)** **100 μg), a growth hormone-releasing peptide 2 (GHRP-2)** **stimulation test (100 μg), an insulin hypoglycemic test (2.5 units of insulin), and a continuous ACTH infusion test (0.5 mg of tetracosactide acetate). Results are shown in Figures 7-11.

The ACTH-cortisol axis showed a lack of responsiveness in both the corticotropin-releasing hormone (CRH) and insulin-induced hypoglycemia tests, but responded to the continuous ACTH infusion, indicating adrenal cortex functionality but deficient endogenous ACTH secretion. In addition to pituitary stimulation tests, serum testosterone was also measured to assess gonadal function. The LH-RH** **stimulation did not show a robust gonadotropin response, but serum testosterone** **was within the normal adult male range (662.4 ng/dL), and the patient had no clinical symptoms of hypogonadism, suggesting preserved gonadal function. TRH, GHRP-2, and insulin tests revealed appropriate responses in the thyroid and growth hormone axes, ruling out deficiencies in these systems.

Based on these findings, the patient was diagnosed with sudden isolated adrenocorticotropic hormone (ACTH) deficiency, classified as secondary adrenal insufficiency. Oral hydrocortisone replacement therapy was initiated at 15 mg/day. Since starting treatment, the patient has had no recurrence of hypoglycemia or impaired consciousness. Levothyroxine sodium hydrate was resumed for his chronic thyroiditis, and his condition remained stable. He is currently working as a cram school instructor and continues outpatient follow-up. The patient received thorough instructions on stress dosing of hydrocortisone, with recommendations to increase the usual maintenance dose by two to three times during periods of physical stress, such as fever or infections, similar to standard management in other forms of adrenal insufficiency. Levothyroxine sodium hydrate was continued at 75 μg/day, which maintained TSH** **and FT4** **levels within the normal range. No further episodes of hypoglycemia or altered consciousness have occurred since initiation of therapy. ACTH and cortisol levels have remained low, consistent with ongoing isolated ACTH deficiency.

## Discussion

Epidemiology

Early cases of isolated ACTH deficiency were first reported by Steinberg et al. in 1954 [5]. With the development of investigation technologies, the number of cases reported has been gradually increasing. It is slightly more common in men, with an average age of onset in the 50s. However, in this case, it was presumed that the patient developed the disease around the age of 24, when fatigue began to appear, so it is regarded as a relatively younger-onset case.

Etiology

The etiology of isolated ACTH deficiency remains incompletely understood. Potential contributing factors include head trauma, lymphocytic hypophysitis, autoimmune hypophysitis (often associated with other autoimmune diseases), certain medications, and radiation therapy for intracranial tumors [6]. In some cases, ACTH-producing cells may be selectively and severely impaired due to organic damage of the pituitary gland from various causes. In this case, selective impairment of ACTH-producing cells was observed, though the underlying cause remains unknown. Notably, this selective impairment occurred within the anterior pituitary, which contains five types of secretory cells. An autoimmune mechanism targeting ACTH-producing cells is considered the most likely etiology. Supporting this hypothesis, anti-pituitary antibodies have been detected in some reported cases [7]. ACTH-secreting cells in the anterior pituitary are particularly susceptible to inflammatory disorders and may be affected in the presence of lymphocytic hypophysitis, although such reports are rare [8].

Additionally, there are cases where ACTH fails to exert its biological activity due to unknown mechanisms. For example, a unique case has been reported in which acquired post-translational processing defects of ACTH precursor proteins led to reduced biological activity [9,10]. In the present case, it is possible that the symptoms of isolated ACTH deficiency were triggered by the initiation of levothyroxine replacement therapy or the onset of infection.

Moreover, in idiopathic isolated ACTH deficiency, coexisting autoimmune endocrine disorders such as chronic thyroiditis are frequently observed, as in the present case [11]. In such patients, even after the diagnosis and treatment of chronic thyroiditis, careful monitoring for symptoms such as fatigue or hypoglycemia is warranted.

Symptoms

The main symptoms consist of general fatigue due to cortisol deficiency, poor appetite, disturbance of consciousness, hypoglycemia, etc. Krude et al. reported a case in which a young woman was transported to the emergency department with impaired consciousness and was subsequently hospitalized due to recurrent hypoglycemia and hyponatremia identified on blood tests, ultimately leading to a diagnosis of idiopathic isolated ACTH deficiency [12]. Similarly, in the present case, comparable laboratory findings were observed. These observations suggest that when such laboratory abnormalities are detected in young patients, clinicians should consider isolated ACTH deficiency in the differential diagnosis and proceed with appropriate evaluation. Furthermore, there are cases in which hypoglycemia or mental decline (such as depression) is misdiagnosed as a mental disorder, so caution is required. Given the fact that the patient in this case had a history of quitting their job due to fatigue, the symptoms of sudden isolated ACTH deficiency were presumed to have appeared around that time.

Examination

The diagnosis of sudden isolated adrenocorticotropic hormone (ACTH) deficiency could be confirmed if the clinical symptoms described above and characteristic examination findings of adrenal insufficiency (low value of ACTH and cortisol in blood, decreased free cortisol in urine, hyponatremia, increased eosinophil granulocyte, etc.) are observed, in addition to the poor response of selective ACTH and cortisol upon the combined administration of four hormones (CRH, GHRH, TRH, and gonadotropin-releasing hormone (GnRH)). The insulin hypoglycemia test is used in combination to differentiate between pituitary and hypothalamic dysfunction.

Treatment

Hormone replacement therapy is similar to that for normal adrenal insufficiency. For adults, a common method is to supplement the body with a fixed amount of cortisol (hydrocortisone), a physiological glucocorticoid. A supplemental dose of 15 mg/day has recently been recommended as the standard dose [13]. As with other replacement therapies for adrenal insufficiency, dosing instructions should be strictly followed in order to increase the amount of supplementation by two- to three-fold during times of stress, such as fever, infection, etc.

Prognosis

As long as a patient takes regular glucocorticoid replacement and the dose is temporarily increased as needed, as described above, the prognosis for life will be good, and they will be able to lead a daily life similar to that of a healthy person.

## Conclusions

We experienced a case presenting with a sudden isolated adrenocorticotropic hormone (ACTH) deficiency, which was diagnosed after repeated episodes of hypoglycemia. The differential diagnosis of this disease should be considered in patients who complain of not only hypoglycemic symptoms but also weight loss and persistent fatigue, as well as patients diagnosed with chronic thyroiditis. Since the prognosis after initiating glucocorticoid replacement therapy is generally good and the patient's quality of life improves, it is important to proactively suspect this disease in order to make an accurate and timely diagnosis.
